# *in vitro* evaluation of mutagenic potential in common oral hygiene products

**DOI:** 10.6026/973206300211510

**Published:** 2025-06-30

**Authors:** Shweta Hegde, Prashanth Shetty, Mridula Nair, Arindam Dalal, Pankaj Bajaj, Sandhya James

**Affiliations:** 1 Department of Oral Medicine and Radiology,Triveni institute of dental sciences, hospital and research centre, Bodri, Bilaspur-495220, Chhattisgarh, India; 2 Department of Biochemistry, Triveni Institute of Dental Sciences, Hospital and Research centre, Bodri, Bilaspur -495220, Chhattisgarh, India; 3 Department of Prosthodontics, crown and bridge and Implantology, Triveni Institute of Dental Sciences, Hospital and Research centre, Bodri, Bilaspur -495220, Chhattisgarh, India

**Keywords:** Mutagenicity, oral hygiene, ames test, mouthwash, toothpaste, *in vitro* assay

## Abstract

The mutagenic potential of five commonly used oral hygiene products using the Ames test is of interest. Fluoridated and herbal toothpastes showed no mutagenicity. Alcohol-based and chlorhexidine mouthwashes demonstrated moderate mutagenic effects, especially with metabolic activation. Hydrogen peroxide mouth rinse exhibited the highest mutagenicity without S9 activation. Thus, the need for further *in vivo* investigation to validate safety profiles is relevant.

## Background:

Oral hygiene products such as toothpastes and mouthwashes are essential components of daily oral care routines, widely used for the prevention of dental caries, gingivitis and halitosis. These products often contain various active ingredients including fluoride, chlorhexidine, alcohol, essential oils, hydrogen peroxide and herbal extracts to enhance their antimicrobial and therapeutic efficacy [1, 2]. While their clinical benefits are well established, concerns have been raised regarding the long-term safety of certain chemical components, particularly with respect to their potential genotoxic and mutagenic effects [3]. Wider surgical margins in oral squamous cell carcinoma are associated with reduced risk of local recurrence, underscoring the importance of adequate margin clearance in treatment [4]. Use of certain oral care products may be linked to a higher risk of developing oral leukoplakia, warranting further research and careful evaluation [5]. This test is particularly valuable for preliminary evaluations due to its simplicity, sensitivity and predictive relevance to *in vivo* conditions [6]. Previous studies have suggested that some oral care formulations, especially those containing alcohol or oxidative agents like hydrogen peroxide, may cause DNA damage or exhibit mutagenic properties under specific conditions [7, 8]. However, the mutagenic potential of several widely used products remains insufficiently explored. Moreover, herbal-based products, although perceived as safer alternatives, may also harbor biologically active compounds capable of genetic alteration [9]. Recent concerns regarding the safety of oral hygiene products have emerged due to reported associations with potentially premalignant oral conditions, such as leukoplakia. Products containing *sanguinarine*, an alkaloid derived from *Sanguinariacanadensis* (bloodroot), have been implicated in the development of leukoplakic lesions, especially in the maxillary vestibule and buccal mucosa [10, 11]. While causality has not been conclusively established, the recurrence of similar clinical presentations in users of *sanguinarine*-based rinses has led to their withdrawal in several countries. These findings highlight the need for rigorous toxicological evaluation of active ingredients in oral care formulations. Moreover, the widespread use of alcohol-containing mouthwashes has led to scrutiny due to the potential production of acetaldehyde, a known carcinogen, in the oral cavity. Acetaldehyde can bind to DNA and form adducts, resulting in mutations that may initiate carcinogenic processes [12]. Although epidemiological evidence remains inconclusive, some studies suggest a dose-dependent risk increase for oral cancers with prolonged use of high-alcohol mouth rinses [13]. Consequently, both regulatory agencies and manufacturers have shown increased interest in reformulating products or providing clearer usage guidelines. In contrast, herbal formulations have gained popularity due to consumer preference for "natural" products. However, the safety of herbal compounds is often under-researched. Plants used in oral care may contain phytochemicals with antimicrobial, anti-inflammatory, or antioxidant properties, but some also possess alkaloids, tannins, or essential oils with cytotoxic or genotoxic potential [14, 15]. Without thorough toxicological profiling, these products may be erroneously assumed to be safe. Thus, evaluating both synthetic and natural ingredients using standardized assays such as the Ames test is essential for a balanced assessment of their mutagenic risks. Given the widespread and prolonged use of these products, it is crucial to assess their genetic safety profile. Therefore, it is of interest to evaluate the mutagenic potential of commonly used oral hygiene products through *in vitro* testing, thereby contributing to a better understanding of their safety implications.

## Materials and Methods:

## Test materials:

Five commonly used oral hygiene products were selected for the study.

These included:

[1] Fluoridated toothpaste

[2] Herbal toothpaste

[3] Alcohol-based mouthwash

[4] Chlorhexidine-based mouthwash

[5] Hydrogen peroxide mouth rinse

All products were obtained from local pharmacies and stored according to the manufacturer's instructions. Serial dilutions were prepared in sterile distilled water prior to testing.

## Bacterial strains and culture conditions:

The mutagenic potential was assessed using the Ames test with *Salmonella typhimurium* strains TA98 and TA100, which are sensitive to frameshift and base-pair substitution mutations, respectively. Cultures were grown overnight in nutrient broth at 37°C with continuous shaking.

## Preparation of S9 Mix:

To simulate mammalian metabolic activation, an S9 mix containing liver enzymes was prepared from Aroclor 1254-induced Sprague Dawley rats. The mix was freshly prepared and used at a concentration of 10% in the test system.

## Ames test procedure:

The plate incorporation method was employed. Each test plate contained 0.1 mL of overnight bacterial culture, 0.1 mL of the diluted test sample, 0.5 mL of phosphate buffer (without S9) or S9 mix (with activation) and 2 mL of molten top agar supplemented with histidine-biotin. The mixture was poured onto minimal glucose agar plates and incubated at 37°C for 48 hours.

## Controls:

Negative control plates received sterile distilled water in place of the test substance. Sodium azide (1 µg/plate for TA100) and 2-nitrofluorene (10 µg/plate for TA98) were used as positive controls.

## Colony counting and data interpretation:

After incubation, revertant colonies were counted manually. A two-fold increase in the number of revertant compared to the negative control was considered indicative of mutagenic activity. All tests were performed in triplicate to ensure reproducibility.

## Statistical analysis:

Mean and standard deviation of revertant colonies were calculated. Statistical comparisons were made using one-way ANOVA followed by Tukey's post hoc test. A p-value < 0.05 was considered statistically significant.

## Results:

The mutagenic potential of five commonly used oral hygiene products was evaluated using the Ames test on *Salmonella typhimurium* strains TA98 and TA100, both with and without metabolic activation (S9 mix). The number of revertant colonies per plate was recorded and compared to negative and positive controls. As shown in Table 1 (see PDF) the fluoridated and herbal toothpastes did not show a significant increase in revertant colonies in either strain, indicating no mutagenic activity. The mean revertant count for fluoridated toothpaste was 24 ± 2 in TA98 and 27 ± 3 in TA100, similar to the negative control values of 22 ± 2 and 26 ± 3 respectively. On the other hand, the alcohol-based mouthwash showed a mild increase in revertants, particularly in TA100 with S9, yielding a mean of 54 ± 4 colonies, which was nearly double the negative control. The chlorhexidine-based mouthwash demonstrated a slightly higher mutagenic response in TA100, reaching 61 ± 5 revertants with S9 activation (Table 1 - see PDF). The hydrogen peroxide-based mouth rinse exhibited the most notable response, particularly in the TA98 strain without S9 activation, with a revertant count of 102 ± 6, significantly exceeding the two-fold threshold compared to the negative control (Table 1 - see PDF). These findings indicate that while most products were non-mutagenic under the conditions tested, the hydrogen peroxide rinse posed a higher mutagenic risk, particularly without metabolic activation. A moderate increase in revertant colonies was observed with alcohol-based and chlorhexidine mouthwashes, especially in strain TA100 with S9 mix.

## Discussion:

This study aimed to assess the mutagenic potential of various commonly used oral hygiene products using the Ames assay, a reliable and widely accepted *in vitro* test for detecting point mutations [1]. The findings revealed that most toothpaste formulations, including fluoridated and herbal variants, did not exhibit mutagenic activity in either TA98 or TA100 strains, consistent with previous studies suggesting their relative safety [2, 3]. Fluoridated toothpaste showed revertant counts comparable to negative controls, indicating that fluoride, at concentrations typically used in dental products, is unlikely to induce mutagenic changes [4]. Herbal toothpaste, often perceived as a natural and safer alternative, also demonstrated non-mutagenicity under the test conditions, aligning with earlier evaluations of plant-based oral products [5, 6]. However, it is worth noting that the safety of herbal constituents can vary based on formulation, concentration and processing [7].

The alcohol-based mouthwash exhibited a moderate increase in revertant colonies, particularly in TA100 strain with S9 metabolic activation. This observation supports existing literature reporting that ethanol, especially in the presence of enzymatic activation, can lead to the formation of acetaldehyde-a compound with known genotoxic properties [8, 9]. Although the mutagenic response remained below that of the positive control, these findings underscore the need for cautious use, especially with prolonged exposure. Chlorhexidine-based mouthwash also showed a similar pattern of mild mutagenic activity. While chlorhexidine is recognized for its strong antimicrobial properties, some *in vitro* studies have reported DNA damage and chromosomal aberrations associated with its use at higher concentrations [10, 11]. Nonetheless, the clinical relevance of such effects remains uncertain due to differences in exposure conditions between laboratory and *in vivo* settings. The most significant finding was the marked increase in revertant colonies caused by the hydrogen peroxide mouth rinse, particularly in TA98 without metabolic activation. Hydrogen peroxide is known to produce reactive oxygen species (ROS), which can induce oxidative stress and DNA strand breaks [12]. Several *in vitro* and animal studies have confirmed its mutagenic and carcinogenic potential at higher doses or with chronic exposure [13, 14]. This highlights the importance of regulating peroxide concentrations in over-the-counter oral products. It is also noteworthy that the mutagenic effects varied between the two bacterial strains used. TA98 is more sensitive to frame shift mutations, while TA100 detects base pair substitutions. The higher revertant counts observed in TA100 for some products suggest that base substitution mutations might be more prominent with these chemical exposures [15]. Despite the informative outcomes, the study has limitations. The Ames test, while useful for initial screening, cannot fully replicate the complex metabolism and defense mechanisms present in human tissues. Thus, further *in vivo* genotoxicity assays and long-term clinical studies are needed to validate these findings.
